# Genome-wide analysis reveals downregulation of miR-379/miR-656 cluster in human cancers

**DOI:** 10.1186/1745-6150-8-10

**Published:** 2013-04-24

**Authors:** Saurabh V Laddha, Subhashree Nayak, Deepanjan Paul, Rajasekhara Reddy, Charu Sharma, Prerana Jha, Manoj Hariharan, Anurag Agrawal, Shantanu Chowdhury, Chitra Sarkar, Arijit Mukhopadhyay

**Affiliations:** 1 CSIR-Institute of Genomics & Integrative Biology, G.N. Ramachandran Knowledge Centre for Genome Informatics, Delhi, India; 2 Department of Pathology, All India Institute of Medical Sciences, Delhi, India; 3 CSIR-Institute of Genomics & Integrative Biology, Genomics & Molecular Medicine, Delhi, India; 4 Premas Biotech Pvt. Ltd, Haryana, India; 5 Mathematics Department, School of Natural Sciences, Shiv Nadar University, Uttar Pradesh, India; 6 CSIR-Institute of Genomics & Integrative Biology, Proteomics & Structural Biology Unit, Delhi, India

**Keywords:** MiRNAs, Cluster, GBM, DLK1-DIO3, MEF2, Tumor Suppressor, Cancer

## Abstract

**Background:**

MicroRNAs (miRNAs) are non-uniformly distributed in genomes and ~30% of the miRNAs in the human genome are clustered. In this study we have focused on the imprinted miRNA cluster miR-379/miR-656 on 14q32.31 (hereafter C14) to test their coordinated function. We have analyzed expression profile of >1000 human miRNAs in >1400 samples representing seven different human tissue types obtained from cancer patients along with matched and unmatched controls.

**Results:**

We found 68% of the miRNAs in this cluster to be significantly downregulated in glioblastoma multiforme (GBM), 61% downregulated in kidney renal clear cell carcinoma (KIRC), 46% in breast invasive carcinoma (BRCA) and 14% in ovarian serous cystadenocarcinoma (OV). On a genome-wide scale C14 miRNAs accounted for 12-30% of the total downregulated miRNAs in different cancers. Pathway enrichment for the predicted targets of C14 miRNA was significant for cancer pathways, especially Glioma (p< 3.77x10^-6^, FDR<0.005). The observed downregulation was confirmed in GBM patients by real-time PCR, where 79% of C14 miRNAs (34/43) showed downregulation. In GBM samples, hypermethylation at C14 locus (p<0.003) and downregulation of *MEF2*, a crucial transcription factor for the cluster was observed which likely contribute to the observed downregulation of the entire miRNA cluster.

**Conclusion:**

We provide compelling evidence that the entire C14 miRNA cluster is a tumor suppressor locus involved in multiple cancers, especially in GBM, and points toward a general mechanism of coordinated function for clustered miRNAs.

**Reviewers:**

Reviewed by: Prof. Gregory J Goodall and Dr. Alexander Max Burroughs

## Background

MicroRNAs are non-randomly distributed across the human genome in clusters where ~30% of them are located within 3 Kb distance from another miRNA [1]. MiRNAs within 50 Kb are reported to be highly correlated in expression across 24 different human organs [2]. Several studies have supported the notion that clustered miRNAs are processed as a single polycistronic transcript [3-6]. Literature evidence indicated that clustered microRNAs are functionally related by targeting the same gene or a group of functionally related genes in the same pathway [7-10]. He *et al.* had proposed the presence of clustered miRNAs to be a pre-requisite for the coordinated control of related biological processes. Their results indicate that non-coding RNAs might act as integral parts of the molecular architecture of oncogene and tumor suppressor networks, establishing the role of oncomiR-1 (mir-17–92 cluster) in lymphomas [11].

One of the largest human miRNA clusters, namely, miR-379/miR-656 on chromosome 14q32.31 [hereafter C14] is encompassed in the conserved imprinted locus DLK1-DIO3 and is unique to the placental mammal lineage with enriched expression in brain [12]. This cluster spanning ~55 Kb on the genome is devoid of protein coding genes as well as repetitive sequences and harbors 52 mature miRNAs. The polycistronic nature of this cluster under positive regulation of Mef2 transcription factor was demonstrated in rat neurons. Mef2 binding site is highly conserved within the mammalian lineage including human [13]. Recently, independent studies comparing genome-wide miRNA expression differences reported both up- and downregulation of individual C14 miRNAs in various human diseases including cancer. While an upregulation was reported for hepatocellular carcinoma [14], downregulation was observed in case of gastrointestinal stromal tumors [15]. Eight miRNAs from C14 were proposed to function as tumor suppressor gene in epithelial ovarian cancer [16]. However, co-ordinated function of these clustered miRNA in human diseases and the plausible underlying mechanism resulting in a cluster-wide deregulation remains unexplored.

Here, we explore the potential role of C14 miRNAs as an essential part of the cellular network and possible underlying mechanisms in human cancers upon its deregulation. Our study revealed that the entire C14 miRNA cluster functions as a potential tumor-suppressor locus in GBM and very likely, in multiple human cancers.

## Results and discussion

Analysis of miRNA expression was performed in 1423 samples from seven cancer types for more than 1000 miRNAs using the available data sets from The Cancer Genome Atlas (TCGA, NIH, USA) on microarray and next generation sequencing platforms. Initial findings were validated by real-time PCR for 112 miRNAs in GBM samples. In addition, mRNA expression profiles and methylation profiles were analyzed for the entire GBM panel available on the TCGA server. To the best of our knowledge this is the largest genomic study establishing the coordinated function of C14 miRNAs.

### The C14 miRNA targets are enriched in genes involved in glioma

**Figure 1:**
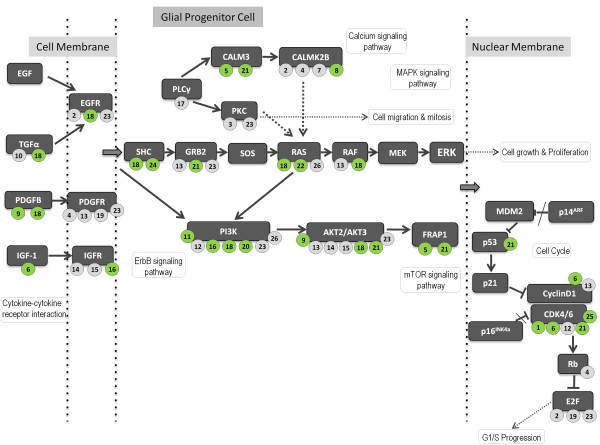
**C14 miRNAs target the glioma pathway.** The biological process has been drawn by adapting the information from the KEGG pathway. mRNA names are written in dark grey boxes. The miRNAs targeting the corresponding genes are denoted by numbers and written inside circles. The miRNAs that were found to be significantly downregulated in GBM (Figure 2) has been colored in green, the ones not differentially expressed are in grey. The miRNAs are numbered according to their respective positions (5’ – 3’ direction) in the genome: hsa-miR-379 (1), -299-3p (2), -380 (3), -1197 (4), -323-3p (5), -323-5p (6), -323b (7), -758 (8), -329 (9), -543 (10), -495 (11), -654-3p (12), -654-5p (13), -376-a1 (14), -376-a2 (15), -539 (16), -382 (17), -134 (18), -668 (19), -485-3p (20), -485-5p (21), -377 (22), -541 (23), -409-3p (24), -409-5p (25), -412 (26).

For each miRNA of C14 we predicted target mRNAs using two independent softwares and their intersection was selected for further studies. These consisted of 28714 predicted target sites for 7944 genes [Additional file 1]. Pathway enrichment of the predicted target genes revealed ‘glioma’ to be one of the most significantly enriched pathway (p<3.77x10^-6^, FDR<0.005) (Figure 1, Additional file 2).

Predicted target genes encompassed 42 out of the previously known 63 glioma genes (http://www.genome.jp/kegg/pathway.html). Out of these, 32 glioma pathway genes are depicted in Figure 1 along with their regulating miRNAs. We observe that 23 genes are targeted by 26 miRNAs from this cluster. The miRNAs significantly downregulated in GBM are marked green on Figure 1. It is intriguing to note that some C14 miRNAs target several genes of Glioma pathway while some Glioma pathway genes are the targets of several C14 miRNAs. For example, miR-134 (#18 in Figure 1), significantly downregulated in GBM, targets eight genes in this pathway according to our analysis. MiR-134 is a brain-specific microRNA and already have proven roles in hippocampal neurons [17], in higher brain functions such as memory formation [18] and also in differentiation of embryonic stem cells [19]. Further research is needed to explore whether miR-134 is one of the crucial (‘hub’) C14 miRNA regulating important biological pathways. Amongst the target genes, majority of them are targeted by more than one miRNA from C14; PI3K and AKT genes are targeted by seven different miRNAs from the cluster. As depicted in the Figure 1, three and four miRNAs out of the seven are significantly downregulated in GBM for AKT and PI3K, respectively. It is likely, that some of these targets are false positives but it is intriguing that ~72% of the genes in this pathway are targeted by 50% of the miRNAs (26/52) from C14.

### The C14 miRNA cluster is downregulated in GBM

**Figure 2:**
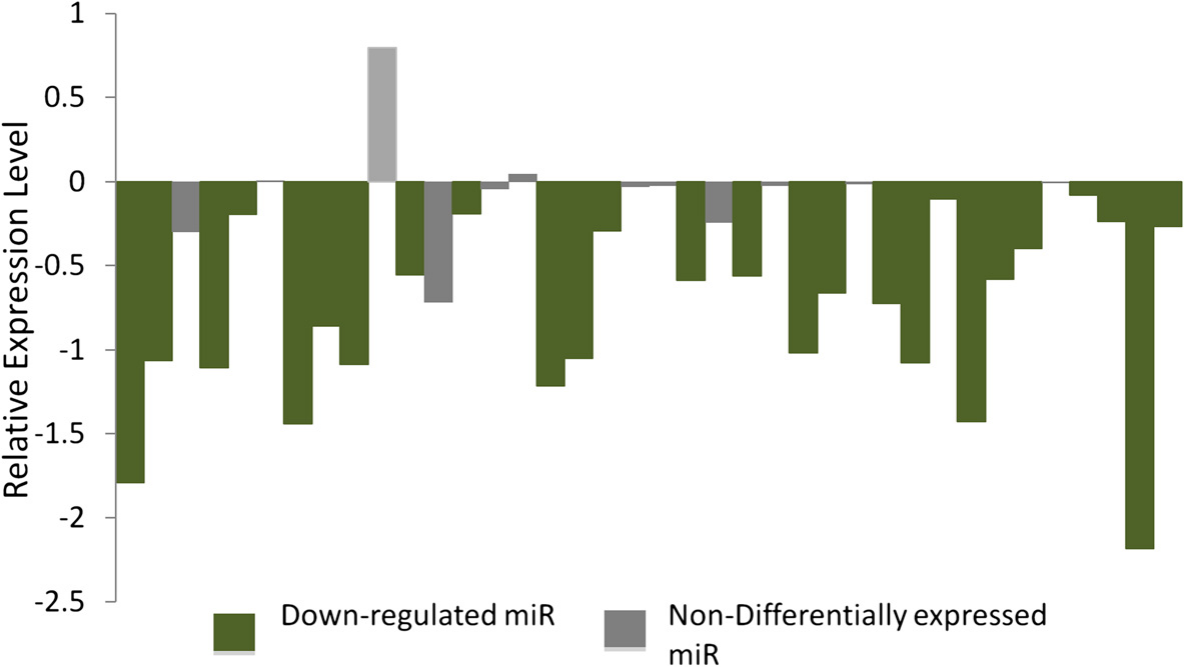
**Downregulation of C14 miRNA cluster in GBM.** On the Y-axis the difference of the median normalized expression levels between GBM and controls (Median_GBM – Median_Control) are plotted for each miRNA (X-axis). Negative values indicate lower expression in GBM. The miRNAs from left to right are, **MiR-379, 411**, 299-3p, **299-5p, 380-3p**, 380-5p, **323, 758, 329,** 494, **495,** 376a, 376a*, 654, 376b, **381, 487b, 539,** 544, 655, **487a,** 382, **134**, 668, **485-3p, 485-5p,** 453 (323b), 154, 154*, 496, 377, 409-3p, 409-5p, 412, **369-3p, 369-5p, 410 and 656**. Green bars indicate significant downregulation in GBM after genome-wide Bonferroni correction (miR names are in bold above). Grey bars indicate expression changes that are not significant post correction. The normalized data were obtained from the TCGA server. Mann-Whitney U test was applied to analyze differential expression. A p value less than 0.05 (post correction) is considered significant. As seen in the figure for the entire cluster (on the horizontal axis) the miRNAs were significantly downregulated in GBM pointing to a coordinated situation.

Genome-wide miRNA expression profile for 534 miRNAs in 496 GBM samples revealed 85 and 95 miRNAs to be downregulated and upregulated, respectively (Additional file 3). Hence this data-set does not show any bias towards a general up- or downregulation of miRNA expression in GBM, in contrast to earlier reports in cancer [20,21]. Out of the 38 miRNAs from C14 (for which data was available), >68% (26/38) showed significant downregulation in GBM patients; none were significantly upregulated (Figure 2). This accounted for 30% of the total downregulated miRNAs (26/85) from the entire genome. To exclude the possibility of this being a chance finding, the analyses were repeated with ten random sets of miRNAs (38 in each set) for the same samples. These miRNAs were chosen excluding the C14 miRNAs. The number of downregulated miRNAs ranged between 3 to 9 out of 38 (95% CI, 4.56-7.03), which were significantly lower than the observation in C14 cluster (26 out of 38, p<10^-10^) (data not shown).

**Figure 3:**
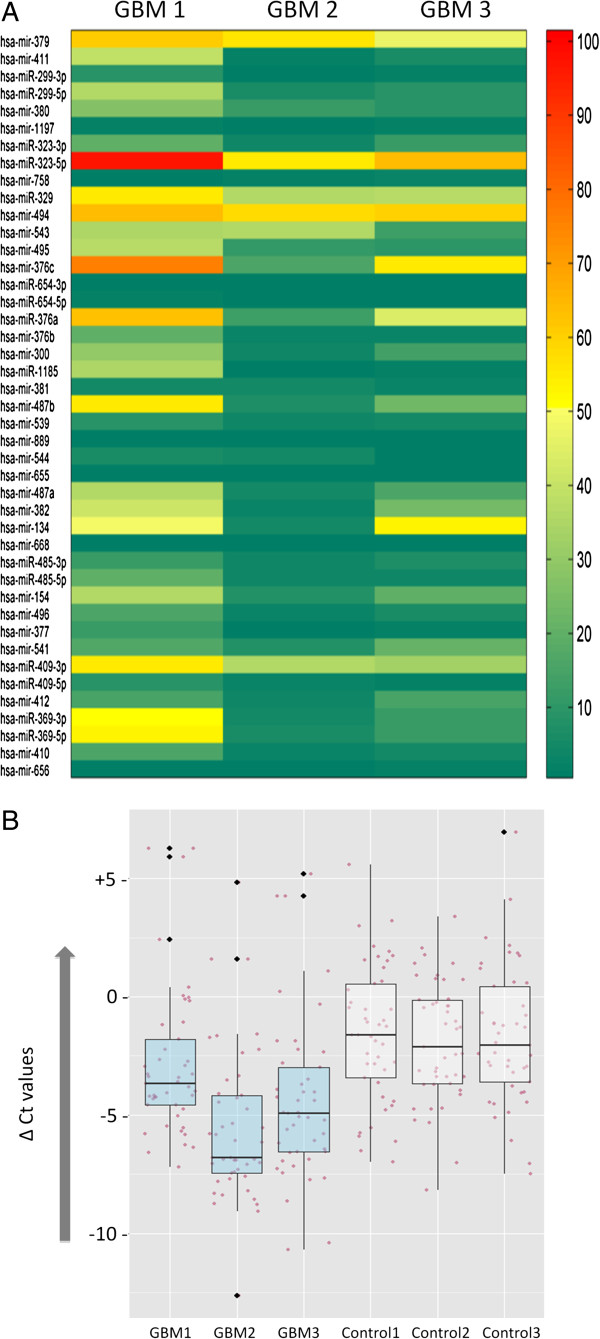
**Real-time PCR validation of C14 downregulation in GBM. A**) The heat map shows the fold changes (calculated by ΔΔCt method) of the respective miRs of C14 in 3 GBM cases with respect to the controls. Fold change <= 0.6 is taken as down-regulation (green) and >=2 is taken as upregulation (red), whereas, the intermediate values are considered to have non-differential expression (yellow) between the samples. As depicted for all the three GBM samples, 79% (34/43) of C14 miRNAs were found to be >40% downregulated in GBM. **B**) The box plots show the median distribution of ΔCt values of the C14 miRs in each of the sample. The GBM samples show distinctive lower median value as compared to the controls. The jitter-plot shows the total data distribution (ΔCt) in each sample.

For experimental validation of the results described above we performed real-time PCR on 43 miRNAs from C14 in brain RNA of GBM patients and controls. As depicted in Figure 3A, the overall expression of tested C14 miRNAs is lower in GBM. Our data shows 79% (34/43) of C14 miRNAs with at least 40% downregulation in GBM (Figure 3B, Additional file 4). To test robustness of our assay we also inspected 69 miRNAs from outside the C14 cluster with previously known altered expression in GBM and produced the expected alteration [22] (both up and down) in 42 of these miRNAs (61%; Additional files 4 and 5). Thus, an independent quantitative estimation of miRNA expression confirmed downregulation of essentially the entire cluster of C14 miRNAs in GBM. The results indicate that majority of the miRNAs in C14 regulate the glioma pathway and a coordinated downregulation of the miRNAs might cause a major systemic perturbation. Whether the observed downregulation is actually a cause of the systemic perturbation or it’s an effect of another global perturbation will be revealed by further studies. It is interesting to note that miR-379 from C14 has four other family members located in the same cluster (http://www.mirbase.org/cgi-bin/mirna_summary.pl?fam=MIPF0000126). Majority of these members were found amongst the downregulated C14 miRNAs in GBM. It remains to be seen whether studying the miRNAs belonging to the same family (may or may not be in the same cluster) can give us more insight into their biology, especially when an additive effect of many miRNAs is investigated.

An earlier study of the corresponding cluster in mice has provided evidence that most, if not all, pre-miRNAs are generated through RNA processing from polycistronic RNA rather than being individually expressed as primary transcripts [23]. Another study demonstrates that this cluster is expressed as a polycistronic unit in rat [13], which corroborates with our aforementioned results showing coordinated downregulation. The C14 miRNAs are reported to have enriched expression in brain [23], which signifies our observed altered expression in GBM, the most aggressive form of brain tumor, which accounts for 52% of all primary brain tumor cases and 20% of all intracranial tumors [MIM: #137800]. Interestingly, the 14q32 locus, encompassing a much larger region of ~20 Mb had been predicted to harbor a tumor suppressor gene in relation to GBM as multiple studies reported loss-of-heterozygosity (LOH) or hypermethylation at this region in glioma patients [24-26]. The probes used in these studies to detect LOH are of a lower resolution and the actual probe site is outside the miRNA cluster. It would be important to carry out future studies by probing the actual cluster region for LOH studies in GBM.

### C14 locus is hypermethylated in GBM

**Figure 4:**
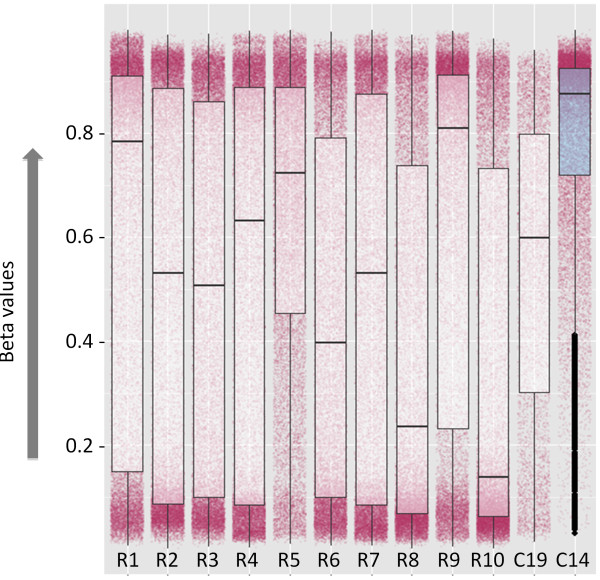
**Hypermethylation of C14 in GBM.** R1 to R10 represent 10 random regions of the genome having similar number of methylation probes as that of C14 (~200 probes). We have used another imprinted microRNA region, present on Chr19, as a control (C19) to test the specificity of the methylation pattern on C14 in GBM. The relative methylation levels are plotted along the vertical axis (beta values). The jitter plots in the background represent the total distribution of the beta-values in each region for all samples. The box plots determine the median beta-values of those regions. C14 is found to be hypermethylated having median beta-value >0.8 (p<0.003), whereas the other sets, including C19 are random in distribution within the normal range (0.2 to 0.8).

Genome-wide methylation status of 76 GBM samples were obtained from the TCGA server (Human Methylation 450K bead chip, Illumina) and tested for the methylation status of the genomic region encompassing the miRNA cluster (~60 Kb) by calculating the beta-values. The Beta-value is the ratio of the methylated probe intensity and the overall intensity (sum of methylated and unmethylated probe intensities). A value of >0.8 and <0.2 is usually considered hyper- and hypo-methylation, respectively [27]. We found that C14 is significantly hypermethylated (p <0.003), having a median beta-value of >0.8 (Figure 4). We have selected a larger imprinted miRNA cluster on chromosome 19 (C19 in Figure 4) as a control region to study methylation in GBM. The methylation at C14 was significantly higher than that in C19. We excluded the possibility that observed hypermethylation of C14 is a random chance by analyzing 10 random regions from the genome. The methylation level of these random regions were within the normal range and comparable with the C19 methylation pattern. As hypermethylation is usually associated with repression of transcription, this can be the causal factor for suppressed expression of the entire cluster in GBM. Genomic coordinates for C14, C19 and 10 random regions are listed in Additional file 6.

### The C14 transcription factor *MEF2* is downregulated in GBM

**Figure 5:**
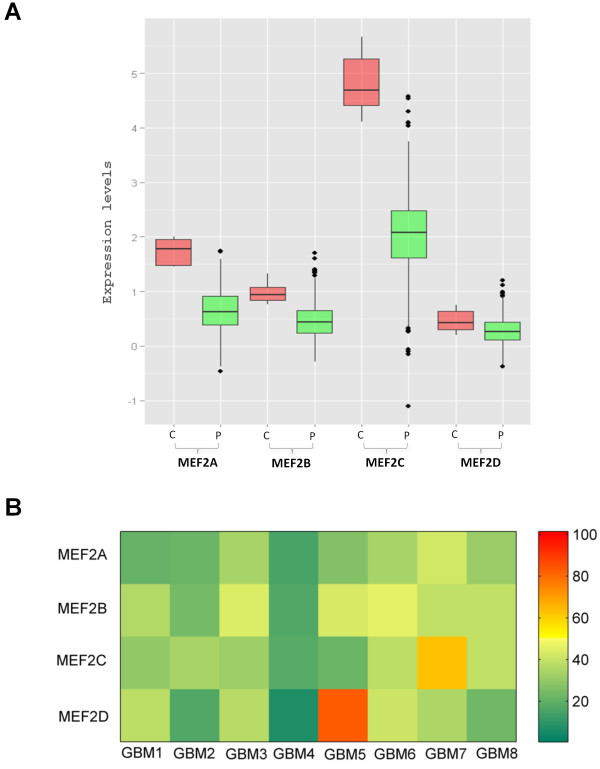
**C14 transcription factor *MEF2 *is downregulated in GBM. A**) Y-axis shows expression levels of different *MEF2* isoforms. “C” and “P” represent controls and patient data, respectively. The median expressions of the different isoforms are distinctly lower in GBM (green) than the control samples (red). The normalized expression values were fetched from the TCGA server. **B**) Real-time PCR validation of *MEF2* expression. Y-axis shows different isoforms of *MEF2* and X-axis shows 8 GBM samples. Fold change <= 0.6 is taken as down-regulation (green) and >=2 is taken as upregulation (red), whereas, the intermediate values are considered to have non-differential expression (yellow) between the samples (relative to data from six controls). Significant downregulation was found for *MEF2A* (p = 0.009) and *MEF2C* (p = 0.04) by Mann-Whitney U test on ΔCt values.

As mentioned in the introduction, Mef2 has been reported as the necessary transcription factor for C14 and hence expression of four family members of *MEF2* was examined in 593 GBM samples (transcriptomic data from TCGA). Significant down-regulation of *MEF2A* and *MEF2C* was found after Bonferroni correction (Figure 5A; two-tailed Mann-Whitney U-test, p values 0.0017 and 0.0011, respectively). Further, we performed real-time PCR analysis of *MEF2* transcripts in eight GBM samples and six controls (Figure 5B) and found significant down-regulation for *MEF2A* (p=0.009) and *MEF2C* (p=0.04).

The hypermethylation of C14 and the downregulation of *MEF2* can be the cause of observed C14 miRNA downregulation in GBM independently or in synergy. As mentioned earlier, Mef2 has been shown to positively regulate transcription of C14 in rat neurons where silencing of Mef2 results in switching off the miRNA expression [13]. MEF2 has an established role in neuronal function and some studies reported its link to cancer. Notch-Mef2 synergistic overexpression results in increasing cellular proliferation and metastasis in drosophila and had higher chance of relapse in human breast cancer patients [28]. Studies in mice have shown Mef2c accelerating myeloid leukemia induced by Sox4 [29]. However, role of MEF2 in human cancers remains largely unexplored and our data indicate that it might have a major role in the pathogenesis, especially in case of GBM.

### C14 miRNAs are downregulated in other human cancers

**Figure 6:**
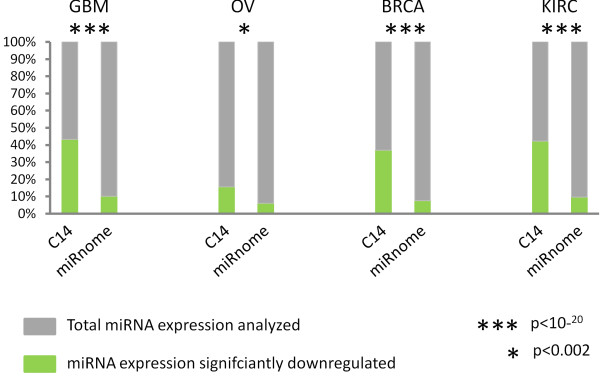
**C14 miRNAs are downregulated in other human cancers.** For each cancer type the total miRNA expression and the number of miRNAs downregulated are shown both for the C14 cluster and the entire miRnome. On the Y-axis the different numbers has been normalized to 100%. The fraction marked in green demarcates the proportion of miRNAs downregulated (significant after Bonferroni correction) against all miRNAs tested (in grey). As depicted in the figure for all the cancer types (albeit less for OV) the proportion of downregulated miRNAs from C14 is much higher than that from the miRnome (green bars). The detailed data and analysis is in Additional file 6. GBM, Gliobalstoma Multiforme; OV, ovarian serous cystadenocarcinoma; BRCA, Breast invasive carcinoma and KIRC, Kidney renal clear cell carcinoma.

We compared the expression of C14 miRNAs in three different cancers excluding GBM, to check the possibility that the downregulation of C14 miRNA cluster is not confined to GBM alone and is general to neoplastic processes. In all cancer types C14 miRNAs accounted for 12-20% of the total downregulated miRNAs (Figure 6 and Additional file 7). We found 61% of C14 miRNAs to be downregulated in KIRC, 46% in BRCA and 14% in OV. This proportion was significantly higher (p<10^-20^ for BRCA, GBM & KIRC and p<0.002 for OV) than expected by random chance (3-5%). These results along with the existing literature strongly suggest that this is a tumor suppressor locus important in general physiology. Our results corroborate the earlier reports wherein deregulation of members of C14 miRNA cluster has been identified in different human diseases. Our study has systematically analyzed the possibility that the entire cluster is in fact functioning like one transcription unit and its deregulation in cancer can be possibly attributed to its altered methylation status and/or repression of transcription factor *MEF2*.

## Conclusion

Our study shows that the entire miRNA cluster miR-379/miR-656 comprising of more than 50 miRNAs is downregulated in multiple human cancers. We have also shown that in GBM, the genomic region harboring this cluster is hypermethylated and the required transcription factor is significantly downregulated. In summary, we propose the entire miR-379/miR-656 cluster on human 14q32 as a single tumor suppressor locus involved in cancer and suggest a general mechanism of coordinated functioning for large polycistronic miRNA clusters.

## Methods

### miRNA target prediction

A local database containing the 3′-UTR sequences for all human genes (NCBI build 36) were made using the publicly available data in ensemble 54 (http://www.ensembl.org/index.html).

To predict targets for each of the 52 mature miRNAs, we have used two independent prediction tools, namely, miRanda (Sep 2008) and TargetScanS 5.1. Default parameters were used for both the tools.

From the two separate lists of predicted targets generated by miRanda and TargetScanS, we have built a consensus list of targets using Perl. We have included a target site only when the genomic co-ordinates were identical in both the predictions. Any 3′-UTR that are predicted targets of the same miRNA in both but at different positions was excluded from the consensus list to reduce false positives.

### Pathway analysis with the target genes

To analyze the enrichment of biological process in the consensus list of target genes, we used the pathway analysis tool DAVID [30,31]. We have used the default parameters, e.g., threshold count 2, modified Fisher’s exact test p-value threshold (EASE score) 0.1. To globally correct enrichment P-values to control family-wide false discovery rate (e.g., <= 0.05), DAVID provides multiple testing correction techniques like Bonferroni, Benjamini and FDR, which were taken into account during the analysis.

### Data mining at the TCGA portal and statistical analyses

The Cancer Genome Atlas (TCGA) data portal (https://tcga-data.nci.nih.gov/tcga/tcgaHome2.jsp) is a comprehensive and coordinated effort to accelerate our understanding of the molecular basis of cancer through the application of genome analysis technologies. All our meta analysis was done using data from the TCGA resource. Below descriptions are given separately for each type of data.

### miRNA expression data and analysis

We have analyzed the genome-wide miRNA expression from microarray data for 1099 samples including GBM (496 GBM + 10 control) and ovarian cancer (585 OV + 8 control) and 324 samples from next generation sequencing of small RNA data which includes Breast invasive carcinoma (BRCA, 80 tumor and 80 matched controls), Kidney renal clear cell carcinoma (KIRC, 67 Tumor and 67 matched controls), Stomach adenocarcinoma (STAD, 9 Tumor and 9 matched controls), Prostate adenocarcinoma (PRAD;3 Tumor and 3 matched controls) and Bladder Urothelial Cancer (BLCA; 3 Tumor and 3 Matched controls). In Additional file 8, the sample IDs for each sample used in the study has been given. For analysis we have used the miRNA expression data generated in Human specific Agilent 8x15k array and Illumina NGS platform. We extracted the quantile normalized log transformed data (level 3) for miRNA expression for all analyses used in this study.

Differential expression of miRNA between patients and controls was determined using non-parametric two tailed Mann-Whitney U test. The test was repeated for each miRNA and corrected for multiple comparisons by multiplying the p value obtained by the total number of miRNAs tested (Bonferroni correction). Identification of upregulated and downregulated miRNAs was determined by similar one-tailed tests. To determine whether the observed patterns of up- or down-regulation was specific to the miRNA cluster tested; we compared the proportions of upregulated, downregulated, and similarly expressed miRNAs from C14 with random sets taken from the genome by chi-square test of proportions. In all cases the p-value threshold was <0.05 (post-correction). For the prostate cancer, stomach adenocarcinoma and the bladder cancer datasets, differential expression was not calculated due to small sample numbers.

### Source of genome-wide methylation data and analysis

We fetched the available raw image files of the GBM methylation data for 76 patients, generated on Infinium HumanMethylation450K BeadChip (Illumina Inc.), from the TCGA Data Portal. The experiment was performed on the DNA samples isolated from the tumor specimen of each patient. For further analyses, we used minfi package of Bioconductor (http://www.bioconductor.org/packages/2.12/bioc/html/minfi.html). This resulted in a matrix of M-values for the corresponding probes. We converted the M-values to beta-values, as beta-values have a more intuitive biological interpretation, using the formula Beta_i_=2^Mi^/(2^Mi^+1) [27]. Due to the absence of control samples, we have used 10 random stretches of the genome and a larger imprinted miRNA cluster on Chr19 (C19), covered by similar number of probes (~200 probes), to rule out the possibility of the observation for C14 being a random occurrence. The beta-values of this locus and 10 other random regions were statistically checked for significant hypermethylation by the application of one tailed t-test. The plots were generated using ggplot2 package of R (http://cran.r-project.org/web/packages/ggplot2/index.html), where the jitter plot showed the total beta-value distribution for each region, whereas, the box plots represented the median values for each region in all the samples.

### Source of transcriptome (mRNA) data

We have also analyzed whole genome mRNA expression data of 593 GBM samples and 10 controls from Agilent G4502A array on the TCGA data portal and extracted expression data which is lowess normalized data (level 3). The downstream analysis was same as described above for the miRNA.

### Sample collection and histopathological analysis

All samples were collected according to the Helsinki Declaration and the ethical review board of All India Institute of Medical Sciences, Delhi, India approved the project. Samples were obtained fresh at the time of surgery and portions were snap frozen in liquid nitrogen and stored at -80°C until use. Rest of the sample was formalin-fixed and paraffin-embedded for routine histopathology. Subsequently, 5 micron sections were cut from the paraffin-embedded tissue and stained by hematoxylin and eosin (H&E) stain. Diagnosis and grading were done as per 2007 WHO classification [32].

Frozen tumor specimens were embedded in freezing medium and 15 serial sections of 40 mm were cut on cryostat and stored immediately in liquid nitrogen cooled vials for RNA isolation. Flanking sections measuring 5 micron were then taken and stained by H&E for histological analysis. The histopathology of each collected specimen was reviewed to confirm adequacy of the sample (i.e., minimal contamination with non-neoplastic elements) and to assess the extent of tumoral necrosis and cellularity. The stored sections were used for nucleic acid isolation only when the flanking H&E sections showed no normal tissue and the tumor content was more than 80% with no or very little necrosis. Along with the tumor specimens for the GBM samples, tissues from six non-GBM samples were also collected to be used as controls. Five of them were patients of grade I transitional meningioma where the supra-tentorial parasagittal sections were collected and one of them suffered from mesial temporal sclerosis where the tissue from temporal lobe was used. In the manuscript these samples has been referred to as controls. For mRNA (MEF2) expression all six controls were used and for miRNA real-time PCR, three of the meningioma samples were used.

### Real-time PCR validation of miRNA and mRNA expression

Total RNA was isolated from brain tissue of GBM patients and controls using the mirVana miRNA Isolation Kit (Ambion, USA) according to the manufacturer’s protocol. The quality and quantity of the RNA was determined by spectrophotometric measurement and gel electrophoresis. cDNA was synthesized from 400 ng total RNA using QuantiMir Kit (System Biosciences, USA), as per the manufacturer’s protocol in a reaction volume of 10 μl.

For real-time quantitative RT-PCR (QPCR), 20x dilutions of the cDNA were made and 1.0 μl from the diluted stock was used for each PCR reaction. Forward primers for 43 microRNAs of the C14 miRNA cluster were designed from the 5′ stem of the mature miRNA and universal reverse primer was used as supplied by the manufacturer (System Biosciences, USA). Relative quantitation was performed using KAPA SYBR® FAST Universal 2X qPCR Master Mix (KAPA BiosystemsInc, USA). PCR reactions were performed on a Light Cycler machine (Roche Lightcycler® 480 II, Roche, USA). Duplicate QPCR reactions were performed for each cDNA to ensure reproducibility.

Brain RNA was used to determine the normal range of expression for the microRNAs. 69 miRNAs having reported differential expression levels of expression in GBM were used as positive controls. The mean expression level of miR-92a was used for normalization [33]. The differences in expression between patients and controls were calculated by using the ΔCT method [34].

Total RNA isolation, quality & quantity check was done exactly same as described above for miRNA. cDNA was synthesized from 1 μg total RNA using High Capacity cDNA Reverse Transcription Kit (Applied Biosystems), as per the manufacturer’s protocol in a reaction volume of 20 μl. For QPCR, 10x dilutions of the cDNA were made and 1.0 μl from the diluted stock was used for each PCR reaction. Forward primers were designed against exon junctions and reverse primer from within the exon. Relative quantitation was performed as before, taking duplicates to ensure reproducibility. The mean expression level of *B2M*[35] was used for normalization. The differences in expression between patients and controls were calculated using the ΔCT method [33]. The primer sequences for each variant are available on request.

## Reviewers’ report

**Title:** Genome-wide analysis reveals down-regulation of miR-379/miR-656 cluster in human cancers

**Versions:** 1 & 2 11 November 2012/ 21 March 2013

**Reviewer number:** 1

**Reviewer:** Prof Gregory J Goodall (nominated by Prof Mark Ragan)

Laddha et al. have profiled publicly available cancer microRNA expression profiles, finding miRNAs of the miR-379/miR-656 cluster to be downregulated in several cancer types, including glioblastoma. Several of these miRs were measured in a small number of glioblastomas by the authors, finding results consistent with the downregulation. Bioinformatic prediction of the targets of members of the cluster indicated an enrichment for genes involved in glioblastoma. Assessment of publicly available gene methylation data indicated the locus is hypermethylated in glioblastomas, consistent with its downregulation. MEF2A was measured in several glioblastomas and found to be reduced relative to controls, consistent with previous reports that MEF2 regulates expression of the locus. Surveying public data for several other cancers suggested downrugulation of the locus may occur in some other cancers.

Comment: The manuscript contains interesting observations regarding miR-379/miR-656 locus in glioblastoma and its likely regulation by MEF2A. The Results section is rather porly structured and explained, and the Figure legends lack detail, requiring constant reference to the Abstract and the Materials and Methods sections to follow what was done and assess the claims. The manuscript would benefit from a rewrite, inserting more information into the Results and Figure legends.

Response: We have incorporated more information in the results section and the figure legends as suggested by the reviewer.

Comment: Figures 2 and 3 shows the downregulation of many members of the cluster in glioblastomas relative to controls, but there is no mention of what the controls are, making interpretation difficult.

*Response: In Figure *2*the normalized (level 3) expression data from TCGA repository has been used to analyze the miRNA expression difference. As mentioned in the ‘miRNA expression data and analysis’ section under ‘Materials and Methods’ the TCGA server provided brain miRNA expression from 10 control samples. In the revised manuscript we have added a supplemental file (supplement 7) with individual samples IDs (TCGA barcodes) for easy reference. As information provided by TCGA these 10 control samples were brain tissues from 10 unrelated individuals who did not suffer from GBM.*

*In Figure *3we have reported data of real-time PCR for samples collected by us. Here the three non-GBM controls were patients of transitional meningioma for whom supratentorial parasagittal sections of the brain were used. This has now been modified in the revised manuscript in the section ‘Sample collection and histopathological analysis’ under ‘Materials and Methods’.

Comment: Page 5 “PI3K and AKT genes are targeted by seven different miRNAs from the cluster” but are these seven miRNAs among the ones that are down-regulated in GBM? Figure 1 would be more useful if it included an indication of which of the C14 cluster miRNAs shown on the Fig are actually down-regulated in GBM.

*Response: We have now modified Figure *1*as suggested by the reviewer. The miRNAs found to be downregulated in GBM have been colored in green. We have also modified relevant sections in the results and the figure legend. As seen in the modified figure, not all seven miRNAs are downregulated as reveled by the statistical analysis of the microarray data. The actual biological cross-talk will be clear after extensive functional studies for mRNA:miRNA interactions.*

Comment: On Page 9 it is stated that cluster miRs were downregulated in several cancers at significantly higher proportion than expected by random chance. The statisitical test used and the P value should be given.

*Response: In all the cancers tested we have found the C14 miRNAs to be downregulated significantly more than expected by random chance (Figure *6*). The p values were: p<10*^*-20*^*for GBM, BRCA and KIRC and p<0.002 for OV. These were obtained by chi-squared tests. The details are in supplemental file 6. We have also added a new figure in the main manuscript with this data (Figure *6*).*

Minor correction

Comment: Page 14. “Flanking sections measuring 5 mm were then taken and stained

by H&E for histological analysis.” Was this not 5 um rather than 5 mM?

Response: We thank the reviewer for pointing out this mistake. It has now been corrected in the revised manuscript.

**Quality of written English:** Acceptable

Reviewer’s response: My comments have been adequately addressed.

Reviewer’s response: The numbering of Figs in the published version will need to match the numbering in the text (currently it does not for Figures 4 onwards).

*Author’s Response: We think the confusion about figure numbering arose from the fact that we have uploaded two separate files for two panels of Figure *3*. So, although the total number of figures are 6, but 7 files have been uploaded for figures. We have checked the text in the manuscript and there are no errors. We hope the two separate panels can be merged at the publication stage.*

Quality of written English: Acceptable

Reviewer's report

**Title:** Genome-wide analysis reveals down-regulation of miR-379/miR-656 cluster in human cancers

**Versions:** 1 & 2 10 December 2012/ 5 March 2013

**Reviewer number:** 2

**Reviewer:** Dr Alexander Max Burroughs (nominated by Dr L Aravind)

Laddha and colleagues mine TCGA data to investigate the role of a specific miRNA-rich genome region in different cancer types. In addition to uncovering a likely role for this genome region in GBM, the authors supply independent experimental data supporting their claims.

Targeted computational analysis of large, publicly-available datasets can provide fruitful avenues of investigation for researchers as the large consortiums generating these datasets often lack sufficient manpower to thoroughly comb through the data. Along these lines, I find the manuscript of general interest particularly since this specific miRNA cluster has not been the subject of extensive research in the past. However, I have a few points for the authors to consider:

Comment: The examined genomic locus covers many miRNA, and several of these belong to the same miRNA family (e.g. miR-379, miR-380, miR-411, miR-758, and miR-1197 all belong to the same family according to miRBase). Is it possible the observed downregulation is largely driven by one or two families being instead of a locus as a whole? I specifically bring this up because of another paper implicating members of the above family in GBM, see Skalsky RL and Cullen BR in published in September 2011 in Plos One.

Response: We thank the reviewer for his concern. Although this may be a concern for other regions of the genome but for the C14 miRNA cluster this issue does not confound our data. We checked the reference suggested by the reviewer and found that they have talked about only one family within the cluster of miR-376 (miR-376a, -b or-c). The other miRs of this cluster are actually not part of the same family. Also upon multiple alignment of the pre-miRNA sequences of the miRNAs from C14 one finds very low similarity between them pointing to the above fact. Although theoretically it is still “possible that the observed downregulation is largely driven by one or two families being instead of a locus as a whole”; but given all the evidences provided by our study this is a remote possibility.

Comment: At several points in the paper the authors compare activity at the C14 locus to randomly-selected genome regions, but explanations of the criteria determining selection of these random regions are relatively scant. In each case are the authors filtering out other miRNA-rich genomic regions or instead selecting for miRNA-rich regions? Are they selecting genome regions with similar characteristics (e.g. similar ratio of coding or non-coding transcripts, similar ratio of repetitive regions, etc.)? If instead the authors are simply taking similarly-sized genomic sequences, I would think that 10 random selections would not be enough to amass a viable background set since localized attributes can substantially influence several of the characteristics being investigated. Since this selection underlies some of the more crucial findings of the manuscript, this could be considered more carefully or at least better-described in the text.

Response: We have used 10 random sets in two different scenarios in this study. Both are separately described below:

*(i) To check that the observed downregulation of C14 miRNAs in cancers is not by chance, we have selected 10 random sets of miRNAs to analyze from the miRNA expression data. This datasets are not from contiguous stretches of genome but consists of similar number of miRNAs as C14 (38 miRNAs randomly selected from genome excluding C14). As the observed downregulation is much more than expected by random chance and we observed significant downregulation in multiple cancers, this finding is unlikely to be a false positive. In addition, as the total number of miRNAs for which expression data was available (for GBM) was only 534, more random sets will actually repeat the same miRNAs across multiple sets making the analyses redundant. The following text in the Results section has more specific information: “****To exclude the possibility of this being a chance finding, the analyses were repeated with ten random sets of miRNAs (38 in each set) for the same samples. These miRNAs were chosen excluding the C14 miRNAs. The number of downregulated miRNAs ranged between 3 to 9 out of 38 (95% CI, 4.56-7.03), which was significantly lower than the observation in C14 cluster (26 out of 38, p<10***^***-10***^***)”.***

(i) To check the possible altered methylation pattern of C14 in GBM, we have compared it with the methylation pattern of another imprinted large miRNA cluster on chromosome 19 (C19). The other 10 regions are randomly selected from the genome. The genomic sizes are not comparable, but the number of methylation probes is comparable (~200 probes per region) in a contiguous genomic stretch. The specific coordinates of each region have been given in the supplementary information. As the distribution of the probes on the microarray is not uniform throughout the genome, it is practically improbable to select enough number of regions with different distributions of mRNAs, miRNAs, repeats etc.). This section has now been modified in the revised manuscript.

Comment: The authors use quite a conservative method for identifying significant miRNA up/down regulation. Did the authors consider a different method, for example edgeR in Bioconductor, and how did these results compare?

Response: edgeR is a tool for analyzing digital gene expression data generated from NGS platforms, taking read counts and library size as inputs. The data we have primarily focused is the GBM dataset, which has microarray data for which edgeR is unsuitable. The other cancer types in TCGA, for which we have used small RNA sequencing data for expression analysis, do not have level 1 data which would have the information required by edgeR for analysis. The different expression data analysis tools primarily differ in their method of normalization of the raw data. As we have used the normalized expression data from TCGA, we have directly applied statistic to test differential expression.

Comment: As currently written, the manuscript is unclear on which cell types were analyzed when examining methylation patterns. Are these the same as the samples used for miRNA profiling? Do they match the same cancer type? Perhaps this is evident to those well-versed with the TGCA dataset but making this clearer would improve readability.

Response: We thank the reviewer for this constructive suggestion. The methylation experiment was performed on the DNA samples isolated from the tumor specimen of each patient. The data were from 76 GBM samples which were also included in the miRNA expression analysis. We have now added a sentence in the relevant section under ‘Materials and Methods’.

Comment: The authors state “the results compel us to say that majority [sic] of the miRNAs in C14 regulate the glioma pathway and a coordinated downregulation of the miRNAs can cause a major systemic perturbation.” I’m not sure the analysis presented by the authors determines causative relationship. It seems quite possible that the opposite could be true: some systemic perturbation is causing the downregulation of the miRNAs in this genomic location.

Response: The statement has been modified as follows: “The results indicate that majority of the miRNAs in C14 regulate the glioma pathway and a coordinated downregulation of the miRNAs might cause a major systemic perturbation. Whether the observed downregulation is actually a cause of the systemic perturbation or it is an effect of another global perturbation will be revealed by further studies.”

Comment: While the authors specifically address upregulation of C14 miRNAs in GBM, how much upregulation is observed in the other cancer lines?

*Response: We actually observed a downregulation (not ‘upregulation’) of C14 miRNAs in GBM and other cancers and all the data presented in the manuscript are from patient tumor samples and not from cancer lines. The proportion of downregulated miRNAs varied from 12-30% of all downregulated miRNAs and all cases were statistically significant. A new figure has been added in the revised manuscript (Figure *6*) to explain the results and the detailed analysis is in Additional file*6*.*

Minor point

Comment: In the conclusions, the authors state “…the required transcription factor is significantly downregulated”. Are the authors certain MEF2 is the only transcription factor active in this location?

Response: Given the complex nature of human biology, it is almost certain that MEF2 is not the only molecule transcriptionally regulating this cluster. However, it has been reported in the literature that regulating Mef2 levels one can regulate the expression levels of the miRNAs in this cluster implicating Mef2 as the most important transcription factor for the cluster. Details of this findings can be found in Fiore R et al, EMBO J.2009; 28:697-710.

**Quality of written English:** Needs some language corrections before being published

Response: We have put in sincere efforts to make the language better and more accurate in the revised manuscript.

Reviewer’s response: The comment was not intended to suggest that all of the miRNAs from C14 belong to the same miRNA family; this is certainly not the case. I was instead making the observation that mir-379 family members (see miRNA gene family mir-379: http://www.mirbase.org/cgi-bin/mirna_summary.pl?fam=MIPF0000126) are present in C14 and appear to largely belong to the set of significantly down-regulated miRNAs; the supplied reference also found members of this family involved in GBM. I was wondering if the authors checked the list of down regulated miRNAs, within and outside of C14, for the presence of complete (or nearly complete) families of miRNAs. While I very much appreciate the author’s detailed responses to the other comments, particularly the extensive clarifications relating to methodology, I think this remains a point of interest: if specific miRNA families are making key contributions this could aid further studies into target identification, throwing more light on GBM.

Author’s Response: We thank the reviewer for clarifying his earlier concern and for providing us information about the family members. In light of his comment we now have included a statement in the re-revised manuscript in the relevant section. The statement is appended below.

*“It is interesting to note that miR-379 from C14 has four other family members located in the same cluster (http://www.mirbase.org/cgi-bin/mirna_summary.pl?fam=MIPF0000126). Majority of these members were found amongst the downregulated C14 miRNAs in GBM. It remains to be seen whether studying the miRNAs belonging to the same family (may or may not be in the same cluster) can give us more insight into their biology, especially when an additive effect of many miRNAs are investigated.”*

Reviewer’s response: I apologize as my initial comment was not very clear: as the authors are surely aware, several different methods exist for addressing the multiple testing problem in both microarray and next-generation sequencing. Use of the Bonferroni correction is a starkly conservative choice; I was interested in why the authors would choose this corrective method over other, more frequently used methods like FDR. Does a more inclusive method increase the total number of genes from other regions in the genome, lessening the “signal” observed from the C14 region?

Author’s Response: As we had used data generated by a third party (TCGA), which is also normalized by them, we wanted to reduce the false positives by using the more stringent Bonferroni correction. However, we understand and appreciate reviewer’s concern and have performed FDR corrections in two of our datasets (namely, GBM & BRCA). We do not observe a significant alteration of the results. Specifically, upon application of FDR the data changes as detailed below:

In case of GBM (microarray platform), the fraction of C14 downregulated miRs vs. miRNome downregulated were 0.763 vs. 0.159 (by Bonferroni) and 0.815 vs. 0.204 (by FDR). In BRCA (NGS platform), the C14 downregulated miRs vs. miRNome downregulated was 0.585 vs. 0.101 (by Bonferroni) and 0.634 vs. 0.154 (by FDR).

For calculating FDR adjusted p values we have run [p.adjust(<file name>, method=”fdr”)] command in R on the p-values obtained from Mann-Whitney test and then taken adjusted p values <0.05.

Since, the change in the data was not altering the conclusion we did not change the dataset presented in the manuscript. This analysis was done to take care of the reviewer’s concern.

Quality of written English: Acceptable

Reviewer’s name: Dr. Yuriy Gusev (reviewer 3)

This reviewer provided no comments for publication.

## Competing interests

None of the authors have any competing interest for the results reported in this study.

## Authors’ contribution

SVL has performed most of the data mining and informatic analysis; SN has performed the RNA isolation, miRNA real-time PCR and analysis of methylation data; DP has performed the real-time PCR of *MEF2* isoforms and also performed the data mining and analysis of *MEF2* expression from TCGA data; RR has established the methylation data analysis pipeline; CS has performed most of the statistical calculations; PJ has helped in sample procurement and storage; MH has helped in the setting up of the miRNA analysis pipeline; AA and SC gave valuable inputs in the statistical and overall focus of the study; CS has clinically characterized the samples included in the study and played lead role as the clinical collaborator; AM has conceptualized and lead this project including arranging for the required funds. The manuscript has been written by AM with significant inputs from SVL, SN and DP. All authors read and approved the final manuscript.

## Supplementary Material

Additional file 1 Predicted targets for C14 miRNA. Click here for file

Additional file 2 Pathway analysis of C14 targets. Click here for file

Additional file 3 Genome-wide miRNA expression analysis in GBM. Click here for file

Additional file 4 Real-time PCR data for miRNA expression in GBM. Click here for file

Additional file 5 Heat map showing differential expression of miRNAs with reported altered expression in GBM. Click here for file

Additional file 6 Genomic coordinates (hg19) of regions analyzed for methylation level in GBM. Click here for file

Additional file 7 Details of miRNA expression analysis in multiple cancers. Click here for file

Additional file 8 TCGA sample IDs included in this study. Click here for file
