# Supplementary figures and images for: Genome-wide analysis reveals downregulation of miR-379/miR-656 cluster in human cancers
Source: Biol Direct. 2013 Apr 24;8:10. doi: 10.1186/1745-6150-8-10 (PMC3680324; doi:10.1186/1745-6150-8-10)

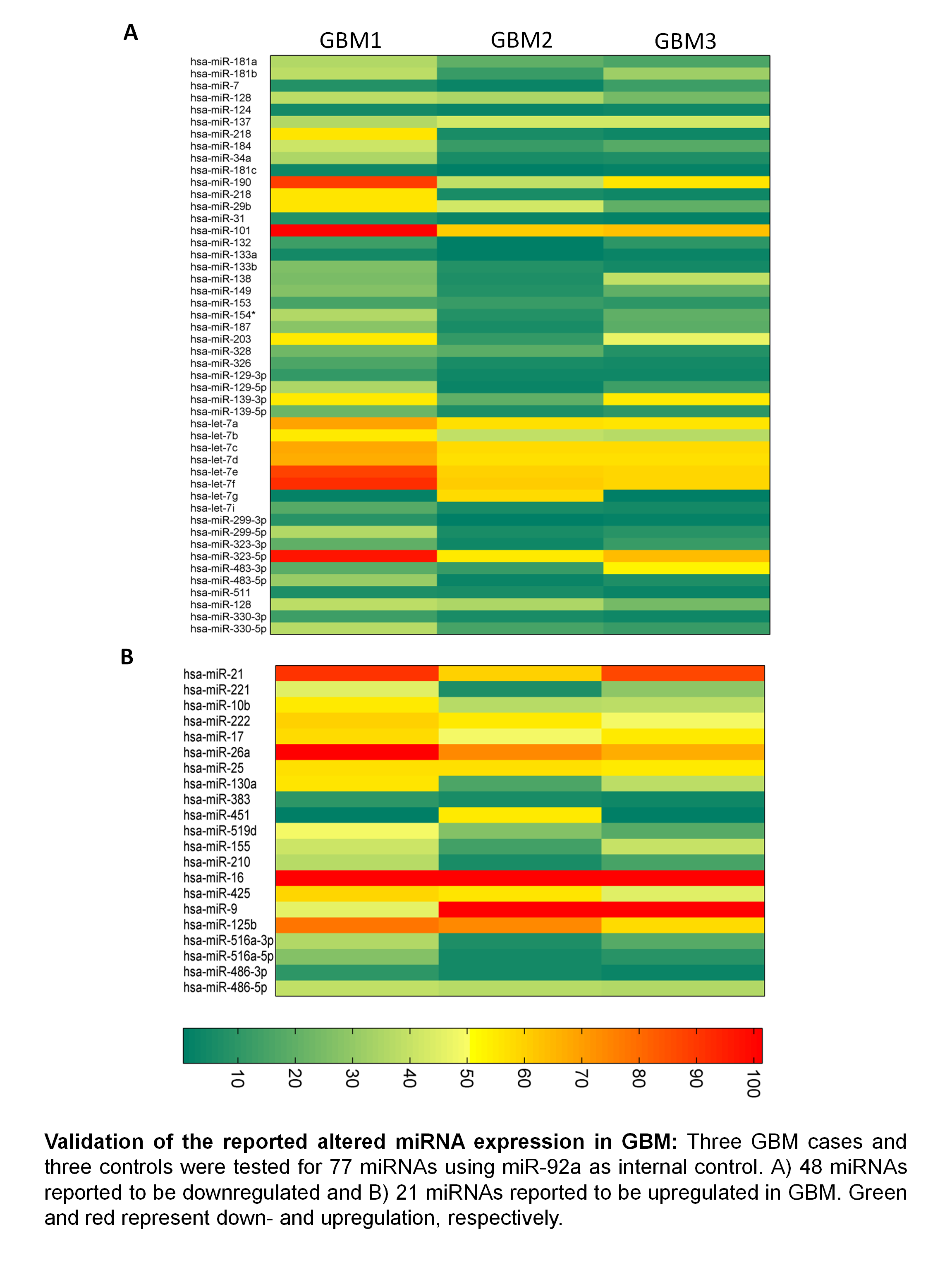

Supplement: Additional file 7 — Details of miRNA expression analysis in multiple cancers. [file 1745-6150-8-10-S7.tiff]
